# Secretion and properties of a hybrid *Kluyveromyces lactis-Aspergillus niger *β-galactosidase

**DOI:** 10.1186/1475-2859-5-41

**Published:** 2006-12-18

**Authors:** Ángel Pereira Rodríguez, Rafael Fernández Leiro, M Cristina Trillo, M Esperanza Cerdán, M Isabel González Siso, Manuel Becerra

**Affiliations:** 1 Departamento de Bioloxía Celular e Molecular, Facultade de Ciencias, Universidade da Coruña, Campus da Zapateira, s/n 15071, A Coruña, Spain

## Abstract

**Background:**

The β-galactosidase from *Kluyveromyces lactis *is a protein of outstanding biotechnological interest in the food industry and milk whey reutilization. However, due to its intracellular nature, its industrial production is limited by the high cost associated to extraction and downstream processing.

The yeast-system is an attractive method for producing many heterologous proteins. The addition of a secretory signal in the recombinant protein is the method of choice to sort it out of the cell, although biotechnological success is not guaranteed. The cell wall acting as a molecular sieve to large molecules, culture conditions and structural determinants present in the protein, all have a decisive role in the overall process.

Protein engineering, combining domains of related proteins, is an alternative to take into account when the task is difficult. In this work, we have constructed and analyzed two hybrid proteins from the β-galactosidase of *K. lactis*, intracellular, and its *Aspergillus niger *homologue that is extracellular. In both, a heterologous signal peptide for secretion was also included at the N-terminus of the recombinant proteins. One of the hybrid proteins obtained has interesting properties for its biotechnological utilization.

**Results:**

The highest levels of intracellular and extracellular β-galactosidase were obtained when the segment corresponding to the five domain of *K. lactis *β-galactosidase was replaced by the corresponding five domain of the *A. niger *β-galactosidase. Taking into account that this replacement may affect other parameters related to the activity or the stability of the hybrid protein, a thoroughly study was performed. Both pH (6.5) and temperature (40°C) for optimum activity differ from values obtained with the native proteins. The stability was higher than the corresponding to the β-galactosidase of *K. lactis *and, unlike this, the activity of the hybrid protein was increased by the presence of Ni^2+^. The affinity for synthetic (ONPG) or natural (lactose) substrates was higher in the hybrid than in the native *K. lactis *β-galactosidase. Finally, a structural-model of the hybrid protein was obtained by homology modelling and the experimentally determined properties of the protein were discussed in relation to it.

**Conclusion:**

A hybrid protein between *K. lactis *and *A. niger *β-galactosidases was constructed that increases the yield of the protein released to the growth medium. Modifications introduced in the construction, besides to improve secretion, conferred to the protein biochemical characteristics of biotechnological interest.

## Background

The enzymatic hydrolysis of lactose by β-galactosidase (E.C. 3.2.1.23) is one of the most promising biotechnological processes in development to use the sugar of the milk whey, a by-product of cheese manufacture with high polluting power [1]. β-galactosidases are widely distributed in nature and are produced by animals, plants and microorganisms (bacteria, fungi and yeast). However, the preparations that are commercially available and rated GRAS come from only a few species of yeast and micro fungi, the most important being *Kluyveromyces lactis *and *K. fragilis*, *Aspergillus niger *and *A. oryzae*. Micro fungi secrete this enzyme extracellularly, however, they produce a lower quantity of enzymatic units than do yeasts and the optimum pH is acid. Micro fungal β-galactosidase utilization for hydrolyzing lactose is restricted to acid wheys [2]. In contrast, yeast β-galactosidase optimum pH is near neutral, consequently making it suitable for saccharifying milk and sweet whey. However, the production and industrial use of this intracellular enzyme are problematic due to the high cost associated with its extraction from the cells and to the low yields obtained as a result of its instability [3].

The secretion of β-galactosidase to the culture medium would facilitate remarkably the downstream processing, eliminating the step of extraction from the cells and reducing the risk of degradation by intracellular proteases. In the case of small peptides or proteins, efficient secretion can be achieved simply by fusing a secretory signal sequence 5' to the gene. However, for large oligomeric proteins of cytosolic origin, like the β-galactosidase of *K. lactis*, [4] consecution of efficient secretion is not so easy. Protein secretion in yeast heterologous systems is influenced by the composition of the medium, culture conditions, phase of growth and structure of the cell wall [5-7]. Protein determinants like size, three-dimensional structure, load, isoelectric point or the glycosylation state are also important [8-10], although their influence has not been completely clarified yet.

Recent studies indicate that the most outstanding structural features influencing secretion are, directly or indirectly, related to protein folding: formation of disulphide bridges [11,12], glycosylation [13,14], and union to BiP [15] or to ubiquitine [12]. Not surprisingly, previous trials of heterologous secretion of β-galactosidase by *S. cerevisiae *rendered levels of 40% of the enzyme in the culture medium in the case of the protein from *A. niger*. This enzyme is extracellular in the micro fungus and therefore suitable structural characteristics for this localization are endogenous. On the contrary, in similar conditions but with the *Escherichia coli *protein, cytosolic in origin, secretion did not surpass 2% in the culture medium [16,17].

In this work, we successfully attempted to convert the intracellular β-galactosidase of *K. lactis *in a protein secreted to the medium. We used engineering techniques based on the construction of hybrid proteins with the extracellular β-galactosidase of *A. niger*. Changes introduced in the hybrid proteins have been evaluated by biochemical methods and discussed to the light of predicted structural models and biotechnological value.

## Results and discussion

### Construction of hybrid enzymes between the intracellular β-galactosidase of *K. lactis *and the extracellular β-galactosidase of *A. niger*

**Figure 1:**
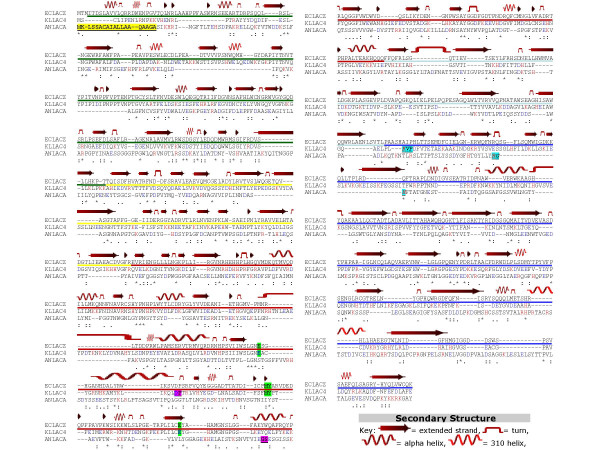
**Amino acid sequence alignment of *E. coli *β-galactosidase with the *K. lactis *and *A. niger *β-galactosidase**. Multiple sequence alignment of *Escherichia coli *β-galactosidase (ECLACZ), *Kluyveromyces lactis *β-galactosidase (KLLAC4) and *Aspergillus niger *β-galacatosidase (ANLACA). "*" means that the residues in that column are identical in all sequences in the alignment. ":" means that conserved substitutions have been observed. "." means that semi-conserved substitutions are observed. Acid (blue colour) and basic (red colour) amino acids of *K. lactis *and *A. niger *β-galactosidase are marked. The coloured bar below the *E. coli *β-galactosidase represents the five different domains structurally determined in the protein (Domain 1: green; Domain 2: yellow; Domain 3: red; Domain 4: light blue; Domain 5: dark blue). The secondary structure of *E. coli *β-galactosidase was obtained from the Protein Data Bank [42]. The localization of the restriction sites *Bam*HI (residues underlined and pink) and *Kpn*I (residues underlined and blue) are indicated. The conserved residues in *E. coli *β-galactosidase and *K. lactis *β-galactosidase important for catalytic function in *E. coli *β-galactosidase are shown in green. The residues of *A. niger *signal sequence are in yellow and underlined.

The extracellular β-galactosidase of *Aspergillus niger *presents, along its primary structure, a lower number of charged amino acids (Figure 1) compared to the intracellular *K. lactis *β-galactosidase, showing the *A. niger *β-galactosidase a 50% reduction in histidine and 43% in lysine content. This difference in charged amino acids could facilitate the secretion of the *A. niger *β-galactosidase, since amino acid charge distribution plays an important role in the localization of secreted and membrane proteins [18].

The construction of hybrid enzymes can be performed by means of different procedures from which new variants are arising constantly [19-23]. In our experimental design, homologous recombination was discarded because the homology between genes was insufficient [24,25]. Therefore, the corresponding constructions were made by PCR amplification of the selected domains, restriction and ligation. Since previous work had demonstrated that, in *K. lactis*, mutant β-galactosidases with large deletions in the N-terminal region were inactive [3] we designed two hybrid proteins between *K. lactis *and *A. niger *β-galactosidases interchanging the C-terminal region. Constructions were made in the pSPGK1 plasmid [26] and were called pSPGK1-LAC4-LACA-*Bam*HI and pSPGK1-LAC4-LACA-*Kpn*I. Both contain in the N-terminus the secretory signal of the pre-sequence of the *K. lactis *killer toxin that has rendered good levels of secretion in other trials [3]. In the first construction, the 500 N-terminal amino acids of the *K. lactis *β-galactosidase were fussed in frame to the 478 amino acids of the C-terminal side of the *A. niger *enzyme. In the second, only the segment corresponding to the fifth domain, 297 amino acids positioned at the C-terminus, of the *K. lactis *β-galactosidase was replaced by the corresponding fifth domain, 274 amino acids positioned at the C-terminus, of the *A. niger *enzyme. The prediction of domains in the proteins from *K. lactis *and *A. niger *(Figure 1) was done by multiple alignments and in comparison with the sequence and structure of the *E. coli *β-galactosidase experimentally determined by crystallography [27].

### Kinetics of secretion

To examine the kinetics of β-galactosidase secretion, a *K. lactis *β-galactosidase mutant strain, MW190-9B, was transformed with the above described constructions and with the plasmid pSPGK1-LAC4, bearing the gene coding for *K. lactis *β-galactosidase, as a control. Discontinuous cultures were made in liquid medium in Erlenmeyer flasks.

**Figure 2:**
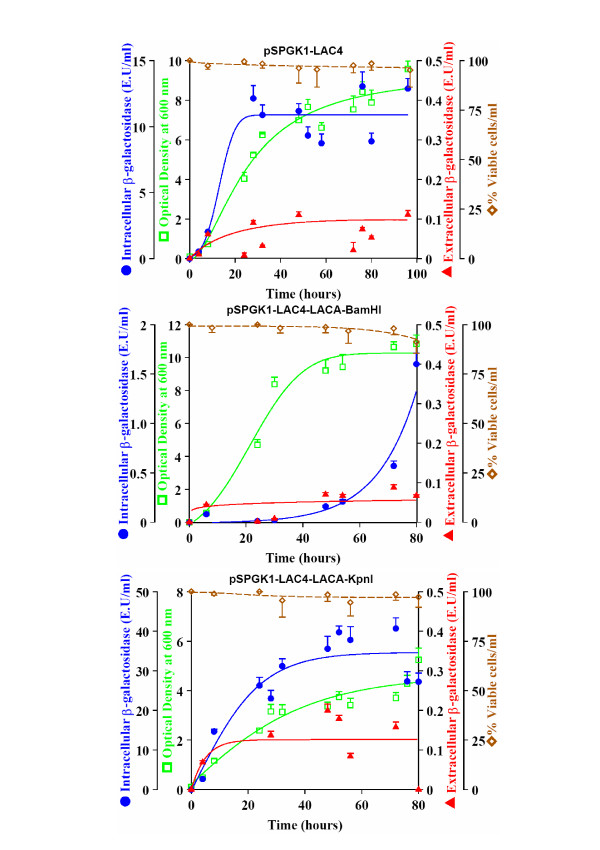
**Kinetics of growth and secretion**. Growth (Optical Density at 600 nm), percentage of viable cells per ml, extracellular and intracellular β-galactosidase production (E. U. mL^-1^) by the MW190-9B strain transformed with the corresponding plasmids. Values represent the mean of 5 different cultures.

The levels of extracellular and intracellular β-galactosidase produced were different in the three transformants (Figure 2). In all cases extracellular β-galactosidase activity was detected in the media. It is important to remark that values of secreted protein are underestimated in this work if compared to other data in the literature. Usually in the bibliography the term extracellular activity includes also the activity of the periplasmic enzyme that is not effectively released to the medium. We have preferred to use the term extracellular to design uniquely the enzyme available in the medium, out of the cell, because its biotechnological use is easier.

The strain transformed with the plasmid pSPGK1-LAC4-LACA-*Bam*HI showed a lower intracellular and extracellular β-galactosidase production than the control. This result may be attributed to the fact that a portion of the catalytic site of the *K. lactis *β-galactosidase was replaced by the catalytic site of *A. niger *β-galactosidase. Nevertheless, MW190-9B transformed with pSPGK1-LAC4-LACA-*Kpn*I showed the highest absolute values of intracellular and extracellular β-galactosidase production, almost three times and twice higher, respectively, than those obtained for MW190-9B transformed with pSPGK1-LAC4, although β-galactosidase activity into the culture medium reaches only 2.6% of the intracellular activity. In this case, the catalytic site from the *K. lactis *enzyme remained intact, since only the segment corresponding to the fifth domain was exchanged.

However, the growth rate of MW190-9B transformed with pSPGK1-LAC4-LACA-*Kpn*I diminished to half of the reached by the strain transformed with pSPGK1-LAC4. Cellular lysis was discarded by measuring cellular viability (Figure 2), therefore this slow growth may be attributed to the fact that the cells direct the available energy towards β-galactosidase production rather than division.

Two conclusions are obtained from these results. First, the C-terminal region of *A. niger *β-galactosidase functionally complements the C-terminal region of *K. lactis *β-galactosidase. Similarly, the fifth domain of the *E. coli *β-galactosidase has been related to the ω-fragment and early studies have shown that it folds independently and complements molecules missing this part of the sequence (ω-complementation) [27]. Second, the construction pSPGK1-LAC4-LACA-*Kpn*I is of biotechnological value and therefore we decide to further characterize this hybrid protein.

### Characterization of the hybrid protein LAC4-LACA-KpnI

Determination of optimum pH and temperature, thermal stability, effects produced by divalent cations upon enzymatic activity and calculation of kinetics constants was performed. To carry out these measures, crude extracts of the strain MW190-9B transformed with pSPGK1-LAC4-LACA-*Kpn*I and with pSPGK1-LAC4 (control) were obtained at the moment of maximum expression of β-galactosidase activity (80 hours).

### Determination of the optimum pH

**Figure 3:**
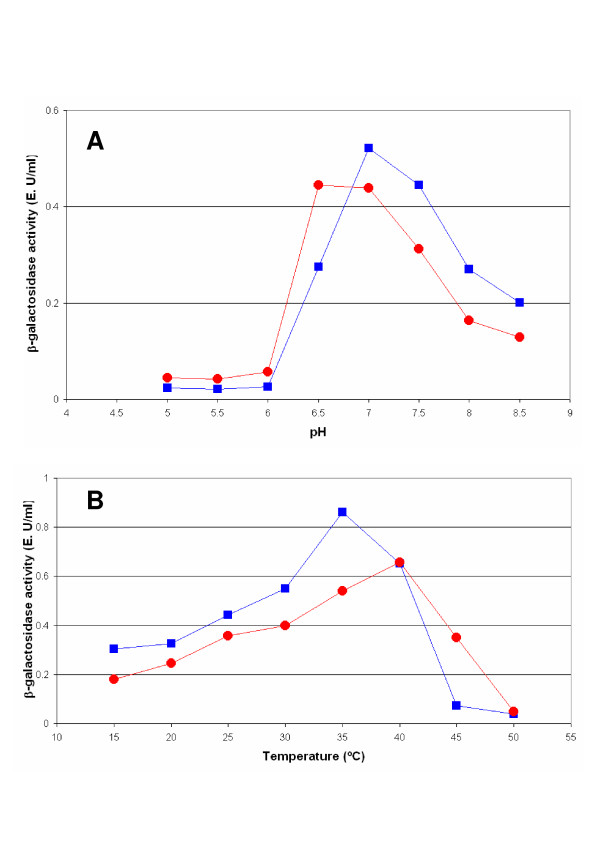
**Determination of the pH and temperature optimum**. Optimum pH (A) and optimum temperature (B) for the hybrid enzyme between the β-galactosidase of *K. lactis *and *A. niger *(red) and the β-galactosidase of *K. lactis *(blue). Experimental variations are less than10% of the value of the point. Data are the mean of three independent experiments.

For the determination of the optimum pH, the measurements of enzymatic activity were carried out in buffer Z aliquots modified to obtain pH values from 5 up to 8.5. As seen in Figure 3A, the optimum pH for the β-galactosidase of *K. lactis *is about 7, whereas for the hybrid protein is slightly acid 6.5. The optimum pH values reported for β-galactosidases from *A. niger *are from 2.5 to 4 [28] whereas from *K. lactis *are from 7 to 7.5 [29,30]. Therefore, the constructed hybrid protein has characteristics with regard to the pH optimum that differs from its precursors. It was reported that, at pH 6.5, the activity of *K. lactis *β-galactosidase decreased significantly due to local changes in charged residues [30]. The hybrid protein, with a different composition in charged amino acids, may buffer these local changes and therefore it may be more tolerant to pH changes during culture.

### Determination of the optimum temperature

The optimum temperature reported for *A. niger *β-galactosidase is 50°C [31] whereas for *K. lactis *β-galactosidase is 30°C [29]. For the determination of the optimum temperature of the hybrid protein and *K. lactis *control, the measurements of enzymatic activity were performed at different temperatures, from 15°C to 50°C (Figure 3B). It was observed that whereas in our conditions the optimum temperature for *K. lactis *β-galactosidase is around 35°C, in the hybrid protein is slightly greater, being near to 40°C. In the same way as for the optimum pH, the constructed hybrid protein presents characteristics that make it more adequate to high temperature during catalysis.

### Thermal stability

**Figure 4:**
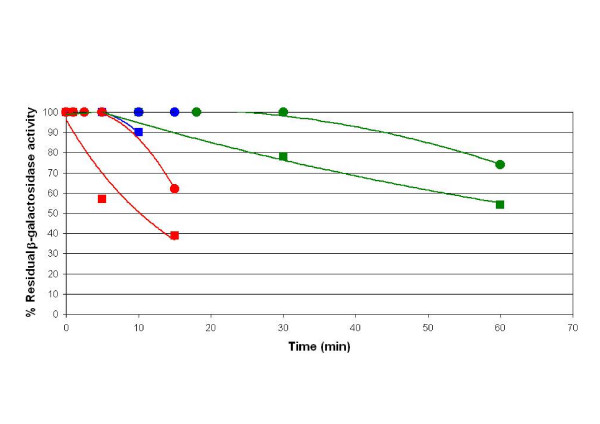
**Determination of the thermal stability**. Determination of the thermal stability at 30°C (green), 42°C (blue) and 50°C (red) for the hybrid enzyme between the β-galactosidase of *K. lactis *and *A. niger *(circles) and the native β-galactosidase of *K. lactis *(square). Experimental variations are less than 10% of the value of the point. Results are the average of two independent experiments.

Thermal stability of the hybrid β-galactosidase was also determined and compared to the native β-galactosidase of *K. lactis*. Before performing the measurement of enzymatic activity, the enzymatic preparation was incubated in buffer Z at different times and temperatures: 30°C, 42°C, 50°C and 60°C. The hybrid β-galactosidase presented a higher stability than the one of *K. lactis *(Figure 4) at all tested temperatures. Almost the 75% of the enzyme kept stable after an hour of incubation at 30°C, the 60% after 15 minutes at 50°C, the 8% after 3 minutes at 60°C (data not show in Figure 4), clearly in advantage to the native *K. lactis *β-galactosidase stability (55%, 40% and 0% respectively). Although biotechnological applications may demand even higher thermal stability of the hybrid β-galactosidase, other procedures exist to improve this factor, i.e. immobilization as previously shown [32].

### Effects of the divalent cations

**Figure 5:**
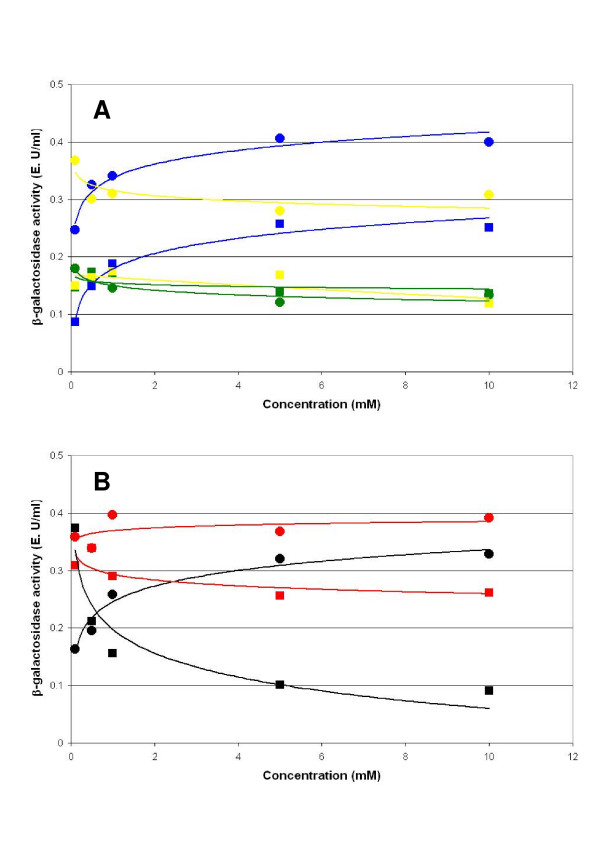
**Determination of the effects of the divalentcations**. Determination of the effects of the divalent cations Mg^2+ ^(blue), Ca^2+ ^(green) and Zn^2+ ^(yellow) (A) and Mn^2+ ^(red) and the Ni^2+ ^(black) (B) on the enzymatic activity of the β-galactosidase hybrid between *K. lactis *and *A. niger *(circles) and the native β-galactosidase of *K. lactis *(square). Experimental variations are less than 10% of the value of the point. Results are the average of two independent experiments.

The activity of *K. lactis *β-galactosidase is stimulated by the presence of some divalent cations, Mg^2+ ^or Mn^2+^, and inhibited by the presence of others, Ca^2+^, Zn^2+ ^and Ni^2+ ^[29,33]. The effect of Mg^2+^, Ca^2+ ^and Zn^2+^on the activity of the hybrid protein and the native *K. lactis *β-galactosidase is similar (Figure 5A). Whereas the presence of Ca^2+ ^or Zn^2+ ^causes a slight inhibition of the activity, Mg^2+ ^stimulates it clearly. Although an increase of the β-galactosidase activity has been described in presence of Mn^2+ ^[29], this stimulatory effect could not be verified in this experiment due to the interference produced by reducing agents present in buffer Z (Figure 5B).

The cation Ni^2+ ^exerts different effects in the activity of the native and hybrid proteins (Figure 5B). As previously described by other authors [33], the cation Ni^2+ ^inhibits *K. lactis *β-galactosidase activity but over the hybrid enzyme the effect is activator. Crystallographic studies identified possible divalent cations binding sites in the structure of the *E. coli *β-galactosidase, although no functional significance was ascribed to them [34]. Further studies to determine the relationship between structural features, cation binding and activity of β-galactosidase will be required.

### Determination of the kinetic constants

**Figure 6:**
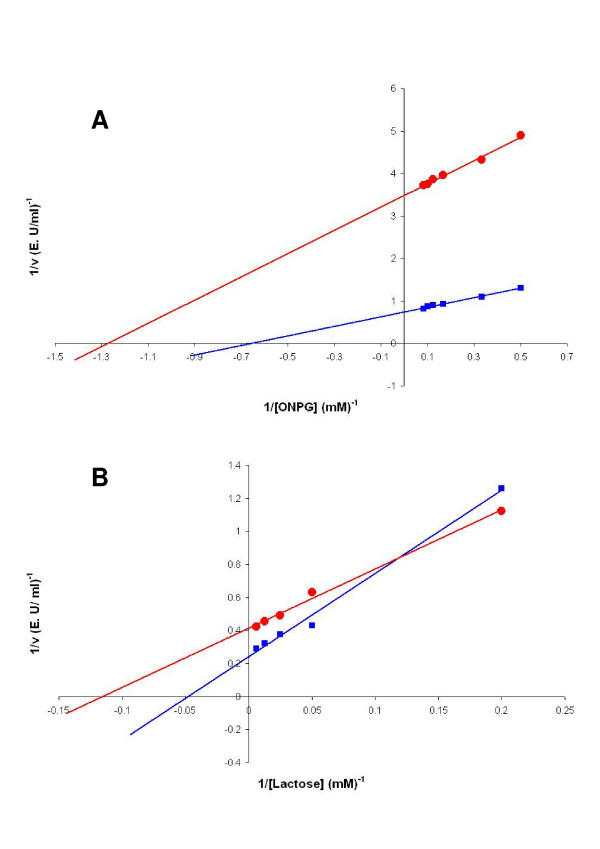
**Lineweaver-Burk plots**. Lineweaver-Burk plot of the reaction catalyzed by the β-galactosidase hybrid between *K. lactis *and *A. niger *(red circles) and the native β-galactosidase of *K. lactis *(square blue) in the presence of the synthetic substrate ONPG (A) or the natural substrate lactose (B). Experimental variations are less than 10% of the value of the point. Results are the average of two independent experiments.

The values of kinetic constants for the hybrid and native β-galactosidases were obtained from double-reciprocal plots (Figure 6). Hybrid β-galactosidase presents a greater affinity both for ONPG (K_m _0.8 mM) and lactose (K_m _8.7 mM) than *K. lactis *β-galactosidase (1.5 mM and 21 mM, respectively). This striking increase in affinity aimed us to look for a structural explanation of the change.

### Prediction of the tertiary structure of the β-galactosidase of *K. lactis *and the hybrid protein

**Figure 7:**
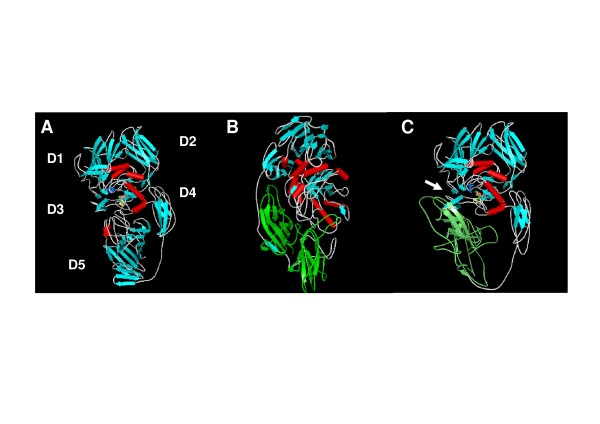
**Ribbon representations**. Ribbon diagram corresponding to the prediction of the tertiary structure of *K. lactis *β-galactosidase (A), *A. niger *β-galactosidase (B) and hybrid β-galactosidase (C) using the Swiss-Model program. The residues mentioned in Figure 1 have been drawn as spheres of colours (E482 blue, M522 green, Y523 yellow, E551 red). D1–D5 identify the five domains of *K. lactis *β-galactosidase (A) predicted by alignment in comparison with the sequence of the *E. coli *β-galactosidase (Figure 1). The fifth domain of the *A. niger *β-galactosidase is coloured in green (B and C). The white arrow (C) shows the slight opening of the third domain in the hybridβ-galactosidase.

Three-dimensional protein structures are important for a detailed understanding of the molecular basis of protein function. In absence of direct experimental data, a computational approach by homology modelling is a reliably method to generate a three-dimensional model for a protein. In order to understand the differences between the hybrid and native *K. lactis *β-galactosidases, a prediction of the tertiary structure of these proteins and the *A. niger *β-galactosidase was made. The server for automated comparative modelling Swiss-Model [35] was used. The amino acids E461, M502, Y503 and E537, considered important residues for the catalytic activity of the *E. coli *β-galactosidase [27,34] and which form the active-site pocket, are highly conserved in the *K. lactis *β-galactosidase (residues E482, M522, Y523 and E551) [36]. As depicted in Figure 7A, a part of the active site is formed by a deep pit that intrudes well into the core of the TIM barrel at the third domain. In addition, there are loops coming from the first and fifth domain. In the case of the hybrid protein (Figure 7C), the fifth domain of the *K. lactis *β-galactosidase was replaced by the corresponding domain of the *A. niger *enzyme (Figure 7B). Structurally, as predicted by the model, this causes a slight opening of the third domain. This could favour the accessibility of the substrate and could explain the change in the kinetic constants.

## Conclusion

The cellular wall represents in yeasts an additional barrier for the excretion of proteins to the culture. The secretory signal directs the proteins across the secretion route up to the periplasmic space but this does not imply that the protein could cross the cell wall. The hybrid protein obtained in this work, by replacing the fifth domain of the β-galactosidase of *K. lactis *by the one of *A. niger*, is active, reaches the culture medium and presents, in addition, greater stability at high temperatures and more convenient kinetics parameters for its biotechnological utilization. Some of these features may be explained to the light of structural changes predicted by homology modelling.

## Methods

### Strains and culture conditions

The *Kluyveromyces lactis *MW 190-9B strain (*MATa lac4-8 uraA Rag+*) was used. Liquid batch cultures of transformed cells were grown in Erlenmeyer flasks filled with 20% volume of culture medium at 250 rpm, unless otherwise stated. As inocula, a suitable volume of a stationary phase culture in complete medium [37] without the amino acid corresponding to the strain auxotrophy, was added to obtain an initial OD_600 _of 0.2. The same medium was also used as culture media. Samples were taken at regular time intervals to measure growth (OD_600_), percentage of viable cells, intracellular and extracellular β-galactosidase activity.

### Vectors and DNA constructions

The pSPGK1-LAC4 [3], a derivative of pSPGK1 plasmid [26] containing the secretory signal that corresponds to the pre-sequence (16 amino acids) of the *K. lactis *killer toxin (α-subunit) and the PCR-amplified *LAC4 *gene (which codes for *K. lactis *β-galactosidase) inserted between the constitutive promoter and the terminator of the *S. cerevisiae *phosphoglycerate-kinase (*PGK*) gene, was used for building new vectors. Vectors were constructed as follows:

-*pSPGK1-LAC4-LACA-BamHI*: plasmid pSPGK1-LAC4 was digested with *Bam*HI. The *Bam*HI-*Bam*HI fragment that contains the C-terminal segment of the *K. lactis *β-galactosidase was removed and replaced by the C-terminal segment of the *Aspergillus niger *β-galactosidase amplified from pVK1.1 [16] with the following oligonucleotides creating *Bam*HI sites on the ends of the PCR product: GAAGGATCCTGAGTCTGGCATCTCG, CCACACCCGTCCTGTGGATCC.

-*pSPGK1-LAC4-LACA-Kpn*I: plasmid pSPGK1-LAC4 was digested with *Kpn*I and ligated to the segment corresponding to the five domain of the *A. niger *β-galactosidase amplified from pVK1.1 with the following oligonucleotides generating *Kpn*I sites on the ends of the PCR product: GCGGTACCCCGCGGACACTTCACCGC, GCGGTACCGCCATCTCCTTGCATGC.

### PCR conditions

A 20 ng amount of template DNA was incubated with 30 pmol of primer-1 and 30 pmol of primer-2 in the presence of 0.25 mM dNTPs, *Taq *or *Pwo *polymerase buffer and 2 U of the corresponding polymerase. Initial denaturation was done at 94°C for 2 min, followed by 30 cycles of 1 min at 95°C, 2 min at 50–57°C and 1.5–2.5 min at 72°C. There was a final incubation at 72°C for 10 min to fill-in ends.

### Molecular biology procedures

*Escherichia coli *DH5a strain (*supE44 DlacU169 f80lacZDM15 hsdR17 recA1 endA1 gyrA96 thi-1 relA1*) was used for the construction of the plasmids and propagation by means of the usual DNA recombinant techniques according to Ausubel *et al*. [38]. Yeast strains were transformed using the lithium acetate procedure [39]. Plasmid uptake and β-galactosidase production by the transformed strains were identified on plates with the chromogenic substrate X-gal in the corresponding auxotrophic medium.

### Percentage of viable cells

The methylene blue solution, which contained 0.01% Methylene Blue (Sigma-Aldrich, M9140) and 2% (w/v) tri-sodium citrate dihydrate in phosphate-buffered saline solution (137 mM NaCl, 2.7 mM KCl, 10 mM Na_2_HPO_4 _and 1.8 mM KH_2_PO_4_, pH 7.4), was mixed with an equal volume of yeast suspension for 10 min. Unstained cells were assumed to be viable. The stained cells in the mixture were quantified under an optical microscope (Nikon Eclipse 50i). The viability of 100 cells, from five replicates of each sample, was assessed and expressed as the mean percentage of viable cells.

### β-galactosidase activity assays

The method of Guarente [40] as previously described [3] was used. One enzyme unit (E. U) was defined as the quantity of enzyme that catalyzes the liberation of 1 μmol of ortho-nitrophenol from ortho-nitrophenyl-β-D-galactopyranoside per min under assay conditions. E.U. are expressed per mL of culture medium.

Throughout this paper and unless otherwise specified, the term extracellular β-galactosidase is used to mean the enzyme in the culture medium and the term intracellular β-galactosidase is used to mean the cell-associated enzyme, both periplasmic and cytoplasmic.

### Preparation of crude protein extracts

For the preparation of crude protein extracts, the cells were harvested by centrifugation at 7000 rpm for 5 min at 4°C and washed once with distilled water. They were suspended in 20 mM Tris-HCl, pH 7.8, 300 mM (NH_4_)_2_SO_4_, 10 mM MgCl_2_, 1 mM EDTA, 10% glycerol buffer with 0.1 mM PMSF, 4 mM Pepstatin, 4 mM Leupeptin and 2 μM β-mercaptoethanol and broken using a sonicator at 16 μm for a total of 20 min at 4°C making four exposures of 5 min, with 5 min intervals after each. Cell debris was removed by centrifugation at 40000 rpm for 90 min at 4°C. The supernatant constituted the cell-free extract.

### Protein determinations

Protein was determined by the method of Bradford [41] using bovine serum albumin (Sigma) as a standard.

### Characterization of the hybrid enzyme

The characterization was carried out from a crude extract of the strain of *K. lactis *MW190-9B/pSPGK1-LAC4-LACA-*Kpn*I obtained at the moment of maximum expression of β-galactosidase activity (80 hours). As a control, in all the essays performed, the same quantity of protein of a crude extract of the strain of *K. lactis *MW190-9B/pSPGK1-LAC4, obtained at the moment of maximum expression of β-galactosidase activity, was taken.

In order to calculate the optimum pH, 60 μg of the crude yeast protein extract were incubated in buffer Z (100 mM Na_2_HPO_4_, 40 mM NaH_2_PO_4_, 10 mM KCl, 1.6 mM MgSO_4 _and 2.7 mL of β-mercaptoethanol for litre of dissolution) adjusted respectively to pH 5; 5.5; 6; 6.5; 7; 7.5 and 8.

For optimum temperature determination, the enzymatic activity of 60 μg of the crude protein extract was measured at different temperatures: 15, 20, 25, 30, 35, 40, 45 and 50°C.

In thermal stability experiments, 30 μg of the yeast crude protein extract were incubated during different periods of time to different temperatures: 30, 42, 50 and 60°C.

For the determination of the effects of divalent cations on the enzymatic activity, 100 μg of the crude protein extract and several increasing concentrations (0.1, 0.5, 1.5 and 10 mM) of CaCl_2_, MgCl_2_, MnCl_2_, ZnSO_4 _or NiCl_2 _were added to the buffer Z and the enzymatic activity was measured as previously described.

### Kinetic studies

The β-galactosidase activity was tested with the artificial substratum ONPG and the natural substratum lactose. The determination of the β-galactosidase activity in 30 μg of the crude extract was made as above explained, but in presence of different concentrations of ONPG: 2, 3, 6, 8 and 12 mM. Alternatively, 60 μg of the crude extract were incubated with different concentrations of lactose: 5, 20, 40, 80 and 160 mM. To determine lactose hydrolysis, a commercial kit was used (*Boehringer-Mannheim*) following the supplier instructions. The method is based on the oxidation of the product D-galactose by the β-galactose dehydrogenase. The amount of NADH formed in this last reaction is stoichiometric to the amount of lactose and D-galactose. The NADH production was measured following the absorbance increase at 340 nm.

### Homology modelling

The models of the *K. lactis *and *A. niger *β-galactosidases and the hybrid protein were made with the fully automated protein structure homology-modelling server Swiss-Model [35].

## Competing interests

The author(s) declare that they have no competing interests.

## Authors' contributions

APR and RFL carried out the experiments for characterization of the hybrid protein and the studies to examine the kinetic of secretion of the transformed strains. MCT participated in the construction of the hybrid proteins. MEC and MIGS participated in the design of the project and discussion of the data and the manuscript. MB conceived of the study and its design and coordinates the analysis of data, homology modelling and drafted the manuscript. All authors have read and approved the final version of the manuscript.

## References

[B1] Becerra M, Rodríguez-Belmonte ME, Cerdán ME, González Siso MI (2004). Engineered autolytic yeast strains secreting *Kluyveromyces lactis *β-galactosidase for production of heterologous proteins in lactose media. J Biotechnol.

[B2] González Siso MI (1996). The biotechnological utilization of cheese whey: a review. Biores Technol.

[B3] Becerra M, Díaz-Prado S, González Siso MI, Cerdán ME (2001). New secretory strategies for *Kluyveromyces lactis *β-galactosidase. Prot Eng.

[B4] Becerra M, Cerdán ME, González Siso MI (1998). Microescale purification of the enzyme β-galactosidase from *Kluyveromyces lactis *reveals that dimeric and tetrameric forms are active. Biotechnol Techniques.

[B5] Rossini D, Porro D, Brambilla L, Venturini M, Ranzi BM, Vanoni M, Porro D (1993). In *Saccharomyces cerevisiae*, protein secretion into the growth medium depends on environmental factors. Yeast.

[B6] Henry A, Masters CL, Beyreuther K, Cappai R (1997). Expression of human amyloid precursor protein ectodomains in *Pichia pastoris*: Analysis of culture conditions, purification and characterization. Protein Expr Purif.

[B7] Wong DW, Batt SB, Lee CC, Robertson GH (2002). Increased expression and secretion of recombinant alpha-amylase in *S. cerevisiae *by using glycerol as the carbon source. J Protein Chem.

[B8] De Nobel JG (1991). Passage of molecules through yeast cell walls: a brief essay-review. Yeast.

[B9] Soo-Wan N, Yoda K, Yamasaki M (1993). Secretion and localization of invertase and inulinase in recombinant *Saccharomyces cerevisiae*. Biotechnol Letters.

[B10] Schuster M, Wassenbauer E, Aversa G, Jungbauer A (2001). Transmembrane-sequence-dependent overexpression and secretion of glycoproteins in *Saccharomyces cerevisiae*. Protein Expres Purif.

[B11] Kowalski JM, Parekh RN, Wittrup KD (1998). Secretion efficieny in *Saccharomyces cerevisiae *of bovine pancreatic trypsin inhibitor mutants lacking disulfide bonds is correlated with thermodynamic stability. Biochemistry.

[B12] Bao WG, Fukuhara H (2001). Secretion of human proteins from yeast: stimulation by duplication of polyubiquitin and protein disulphide isomerase genes in *Kluyveromyces lactis*. Gene.

[B13] Sagt CMJ, Kleizen B, Verwaal R, De Jong MDM, Müller WH, Smits A, Visser C, Boonstra J, Verkleij AJ, Verrips CT (2000). Introduction of an N-glycosylation site increases secretion of heterologous proteins in yeasts. Appl Environ Microbiol.

[B14] Lee J, Park JS, Moon JY, Kim KY, Moon HM (2003). The influence of glycosylation on secretion, stability, and immunogenicity of recombinant HBV pre-S antigen synthesized in *Saccharomyces cerevisiae*. Biochem Biophys Res Commun.

[B15] Katakura Y, Ametani A, Totsuka M, Nagafuchi S, Kaminogawa S (1999). Accelerated secretion of mutant beta-lactoglobulin in *Saccharomyces cerevisiae *resulting from a single amino acid substitution. Biochim Biophys Acta.

[B16] Kumar V, Ramakrishnan S, Teeri TT, Knowles JKC, Hartley BS (1992). *Saccharomyces cerevisiae *cells secreting an *Aspergillus niger *beta-galactosidase grow on whey permeate. Bio/Technol.

[B17] Pignatelli R, Vai M, Alberghina L, Popolo L (1998). Expression and secretion of beta-galactosidase in *Saccharomyces cerevisiae *using the signal sequences of GgpI, the mayor yeast glycosylphosphatidylinositol-containing protein. Biotechnol Appl Biochem.

[B18] Boyd D, Beckwith J (1990). The role of charged aminoacids in the localization of secreted and membrane proteins. Cell.

[B19] Nixon AE, Ostermeier M, Benkovic SJ (1998). Hybrid enzymes: manipulating enzyme design. Trends Biotechnol.

[B20] Chen R (1999). A general strategy for enzyme engineering. Trends Biotechnol.

[B21] Kikuchi M, Harayama S (2002). DNA shuffling and family shuffling for *in vitro *gene evolution. Methods Mol Biol.

[B22] Lutz S, Patrick WM (2004). Novel methods for directed evolution of enzymes: quality, not quantity. Curr Opin Biotechnol.

[B23] Bloom JD, Meyer MM, Meinhold P, Otey CR, MacMillan D, Arnold FH (2005). Evolving strategies for enzyme engineering. Curr Opin Struct Biol.

[B24] Crameri A, Raillard SA, Bermúdez E, Stemmer WP (1998). DNA shuffling of a family of genes from diverse species accelerates directed evolution. Nature.

[B25] Harayama S (1998). Artificial evolution by DNA shuffling. Trends Biotechnol.

[B26] Fleer R, Chen XJ, Amellal N, Yeh P, Fournier A, Guinet F, Gault N, Faucher B, Folliard F, Fukuhara H, Mayaux JF (1991). High-level secretion of correctly processed recombinant human interleukin-1β in *Kluyveromyces lactis*. Gene.

[B27] Jacobson RH, Zhan XJ, DuBose RF, Matthews BW (1994). Three-dimensional structure of β-galactosidase from *E. coli*. Nature.

[B28] Widmer J, Leuba JL (1979). Beta-Galactosidase from *Aspergillus niger*. Separation and characterization of three multiple forms. Eur J Biochem.

[B29] Dickson RC, Dickson LR, Markin JS (1979). Purification and properties of an inducible β-galactosidase isolated from the yeast *Kluyveromyces lactis*. J Bacteriol.

[B30] Tello-Solís SR, Jiménez-Guzmán J, Sarabia-Leos C, Gómez-Ruíz L, Cruz-Guerrero AE, Rodríguez-Serrano GM, García-Garibay M (2005). Determination of the secondary structure of *Kluyveromyces lactis *β-galactosidase by circular dichroism and its structure-activity relationship as a function of the pH. J Agric Food Chem.

[B31] Santos A, Ladero M, García-Ochoa F (1998). Kinetic modelling of lactose hydrolysis by a β-galactosidase from *Kluyveromyces fragilis*. Enzyme Microb Technol.

[B32] Makowski K, Bialkowska A, Szczesna-Antczak M, Kalinowska H, Kur J, Cieslinski H, Turkiewicz M (2006). Immobilized preparation of cold-adapted and halotolerant Antarctic β-galactosidase as a highly stable catalyst in lactose hydrolysis. FEMS Microb Ecol.

[B33] Kim SH, Lim KP, Kim HS (1997). Differences in the hydrolysis of lactose and other substrates by β-D-galactosidase from *Kluyveromyces lactis*. J Dairy Sci.

[B34] Matthews BW (2005). The structure of *E. coli *β-galactosidase. C R Biologies.

[B35] Swiss-Model. http://swissmodel.expasy.org//.

[B36] Poch O, L'Hote H, Dallery V, Debeaux F, Fleer R, Sodoyer R (1992). Sequence of the *Kluyveromyces lactis *β-galactosidase: Comparison with prokaryotic enzymes and secondary structure analysis. Gene.

[B37] Zitomer RS, Hall BD (1976). Yeast cytocrome c messenger RNA in vitro translation and specific inmunoprecipitation of the *CYC1 *gene product. J Biol Chem.

[B38] Ausubel FM, Brent R, Kinston RE, Moore DD, Seidman JG, Smith JA, Struhl K (1995). Current Protocols in Molecular Biology.

[B39] Ito H, Fukuda Y, Murata K, Kimura A (1983). Transformation of intact yeast cells treated with alkali cations. J Bacteriol.

[B40] Guarente L (1983). Yeast promoters and *lacZ *fusions designed to study expression of cloned genes in yeast. Meth Enzymol.

[B41] Bradford MM (1976). A rapid and sensitive method for the quantification of microgram quantities of protein utilizing the principle of protein-dye binding. Anal Biochem.

[B42] Protein Data Bank. http://www.pdb.org/pdb/home/home.do.

